# Dynamic generation and modulation of acoustic bottle-beams by metasurfaces

**DOI:** 10.1038/s41598-018-31066-5

**Published:** 2018-08-23

**Authors:** Di-Chao Chen, Xing-Feng Zhu, Qi Wei, Da-Jian Wu, Xiao-Jun Liu

**Affiliations:** 1 Jiangsu Key Laboratory of Opto-Electronic Technology, School of Physics and Technology, Nanjing Normal University, Nanjing, 210023 China; 2 Key Laboratory of Modern Acoustics, Department of Physics, Nanjing University, Nanjing, 210093 China

## Abstract

Acoustic bottle-beams have been realized by acoustic metasurfaces (AMs) composed of space-coiling subunits. By manipulating the transmitted acoustical phase, the special AM can generate two intersecting accelerating beams along the designed convex trajectories, forming the acoustic bottle-beam. The transmitted acoustic bottle-beams are investigated theoretically and demonstrated numerically. We find that the shape and area of the acoustic bottle-beam could be statically controlled by designing the AM as well as dynamically modulated by the incident angles. In addition, the highly efficient acoustic focusing could be obtained at the convergence point of the bottle-beams, which also could be adjusted dynamically by the incident angles. It is further found that this focusing is robust against the obstacle scattering. The realization and manipulation of acoustic bottle-beams may have potential applications in biomedical imaging/therapy and non-destructive evaluation.

## Introduction

Bottle-beam has become a subject of immense interest in the past decade because of their attractive fundamentals and applications^1–8^. Initially, many efforts have been devoted into optical bottle-beams, which were mainly used in optical tweezers for trapping and manipulating small particles^9–13^. Inspired by optical bottle-beams, acoustic bottle-beams have also received increasing attention due to their potential applications in micro-particle manipulation, medical ultrasound, and ultrasonic imaging^14–16^. Up to now, the generations of the acoustic bottle-beams depend on the transducer arrays^14,15^. However, the bulky size and sophisticated configuration of the transducer design hamper their further applications.

The emergence of metasurfaces (artificially engineered surfaces comprising phase shifters) provides a new way to manipulate wavefront freely^17–19^. In particular, the metasurface can be fashioned into a compact planar profile with subwavelength thickness, thereby reducing the size of acoustic device. Many novel acoustic phenomena including negative extraordinary reflection/refraction^20^, diffuse reflection^21^, focusing^22–24^, and beam steering^25–27^ have been realized based on acoustic metasurfaces (AMs). In this work, we achieve the acoustic bottle-beam using well-designed AMs. A recent proposed three-layer acoustic space-coiling (TAS) structure is used as subunits to build the AM^28^ and the thickness of acoustic bottle-beam generator can be reduced down to 0.22*λ*. The acoustic field distributions of the acoustic bottle-beams have been demonstrated numerically using finite element method (FEM). It is found that the shape and area of the bottle-beams can be dynamically controlled by adjusting the geometry of the space-coiling subunit or the incident angle. In addition, we further study the focus performance of the generated acoustic bottle-beams.

## Results

**Figure 1:**
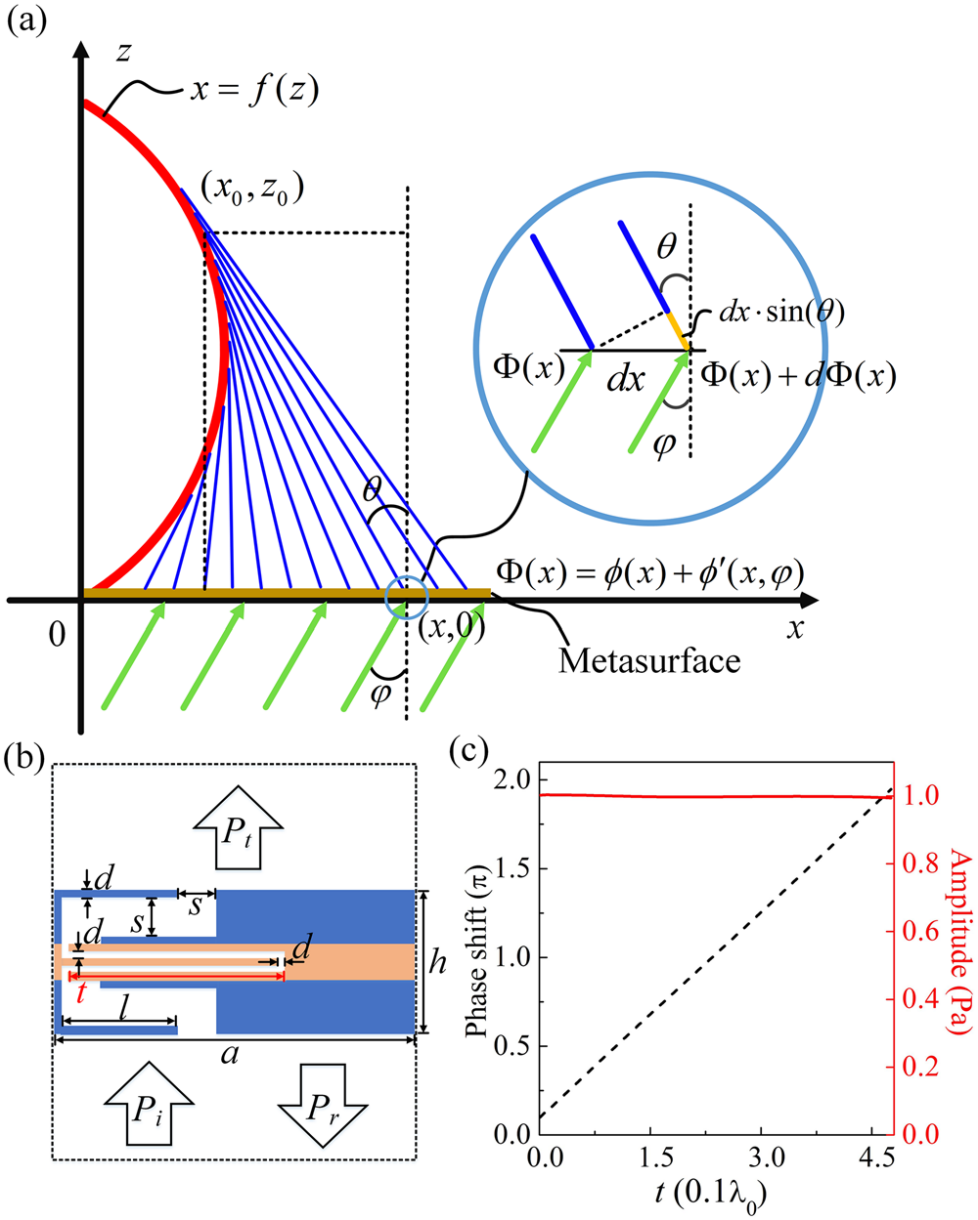
Illustration of an arbitrary convex trajectory *x* = *f*(*z*) (red line), the tangent line (blue lines) and the AM (brown line). ${\rm{\Phi }}(x)=\varphi (x)+\varphi ^{\prime} (x,\phi )$ is the spatial phase profile in the *x*-axis, where *ϕ*(*x*) is the phase shift caused by the AM and $\varphi ^{\prime} (x,\phi )$ is the phase shift caused by the incident angle *φ*. *x*_0,_*z*_0_ is a point on the trajectory and *θ* is the angle between the *z*-axis and the tangent line through the point *x*_0,_*z*_0_. (**b**) Schematic of a three-layered acoustic space-coiling subunit with a variable *t*. (**c**) Relative phase shift (the dashed line for the left-hand scale) and transmissivity (solid line for the right-hand scale) of the three-layered acoustic space-coiling subunit as a function of *t*.

We consider the coordinate system depicted in Fig. 1(a). An acoustic plane wave propagates from the down side into the AM (brown line) placed in the *x*-axis. *x* = *f*(*z*) denotes an arbitrary designed trajectory (red line), which will be realized by the transmitted acoustic waves with the spatial phase profiles through the AM. The spatial phase profile Φ(*x*) can be expressed as

1 $${\rm{\Phi }}(x)=\varphi (x)+\varphi ^{\prime} (x,\phi ),$$

where *ϕ*(*x*) is the phase shift caused by the AM and $\varphi ^{\prime} (x,\phi )$ is the phase shift due to the incident angle *φ*. When an acoustic plane wave normally impinges on the AM, $\varphi ^{\prime} (x,\phi )=0$. Suppose that (*x*_0_, *z*_0_) is a point on the trajectory and *θ* is the angle between the *z*-axis and the tangent line (blue line) through the point (*x*_0_,*z*_0_). The cross point of the tangent line and the *x*-axis is (*x*, 0). According to the Fermat’s principle^29^, the derivative of the phase accumulated along the actual trajectory should be zero with respect to infinitesimal variations of the path. The phase relation depicted in the light blue circle in Fig. 1(a) can be described as

2 $${\rm{\Phi }}(x)+d{\rm{\Phi }}(x)+k\cdot dx\cdot \,\sin (\theta )={\rm{\Phi }}(x),$$

where *d*Φ(*x*) represents the phase shift, *dx* is the infinitesimal distance between two cross points along the *x* direction, and *k* is the wavenumber. Then, the relation between the spatial phase profile Φ(*x*) and the angle *θ* can be deduced as

3 $$\frac{d{\rm{\Phi }}(x)}{dx}=-\,k\,\sin (\theta )\Rightarrow \frac{d\varphi (x)}{dx}+\frac{d\varphi ^{\prime} (x,\phi )}{dx}=-\,k\frac{\tan (\theta )}{\sqrt{1+{\tan }^{2}(\theta )}}.$$

The slope of the tangent line $\tan (\theta )=-\,f^{\prime} ({z}_{0})$ and $f^{\prime} ({z}_{0})$ is the first order derivative of the trajectory. The intercept of the tangent line on the *x*-axis can be obtained as

4 $$x={x}_{0}-{z}_{0}f^{\prime} ({z}_{0})=f({z}_{0})-{z}_{0}\,f^{\prime} ({z}_{0}).$$

Based on Eqs (3) and (4), the curve *x* = *f*(*z*) can be viewed as an envelope to a family of tangents (blue lines) that relate each point (*x*_0_,*z*_0_) on the curve to the point (*x*, 0) on the boundary^30–32^. We could obtain the arbitrary trajectory by adjusting phase shifts of the AM and the incident angle *φ*.

Figure 1(b) show the structure of a TAS subunit, which is used to establish the AM. To achieve the acoustic bottle-beam, the subunits of the AM must cover a complete 2*π* of phase change with high transmission amplitudes. In our model, for simplicity, only one parameter (length *t* of horizontal bar) is picked to adjust the transmitted phase shift and amplitude through the TAS subunit. Some of the other structural parameters, such as *d* and *s*, also could modify the transmitted phase shift and amplitude. But the modulations of *d* and *s* on the transmitted phase shift and amplitude through the TAS subunit cannot meet the requirements for the bottle-beam simultaneously. Throughout this work, the working frequency is fixed at 3.432 kHz, *s* = 0.065*λ*_0_, *d* = 0.01*λ*_0_, *l* = 0.1538*λ*_0_, *a* = 0.5*λ*_0_, and *h* = 0.22*λ*_0_. Figure 1(c) shows the transmitted phase shifts (solid line for left-hand scale) and amplitudes (dashed line for right-hand scale) of the acoustic plane wave through the TAS subunits as a function of *t*. The amplitude of the incident plane wave is fixed at 1 Pa. It is observed with increasing *t*-value that the phase shift changes almost linearly from 0 to 2*π* while the transmission amplitude keeps a very high value. Therefore, this space-coiling AM could manipulate the phase of the acoustic waves flexibly and efficiently.

The acoustic bottle beam encloses a region with close-to-zero sound pressure surrounded by higher acoustic pressure fields^8^. To generate an arbitrary acoustic bottle-beam, an AM with two equidistant spatial phase profiles is proposed to realize two intersecting self-accelerating beams based on TAS subunits. We first investigate a circular acoustic bottle-beam based on the AM with symmetric phase profiles in two sides of *x*-axis. The trajectory is set as a circle with the radius of *a*, centered at point (0, *a*). This circle is represented as $f(z)=\pm \,\sqrt{{a}^{2}-{(z-a)}^{2}}$. If the incident angle *φ* = 0, $\varphi ^{\prime} (x,\phi )=0$. The corresponding spatial phase profile on the *x*-axis could be obtained based on the caustic theory and geometrical properties^30,31^

5 $${\rm{\Phi }}(x)=\varphi (x)=-\,k[|x|-2a\,\arctan (\frac{|x|}{a})],$$

**Figure 2:**
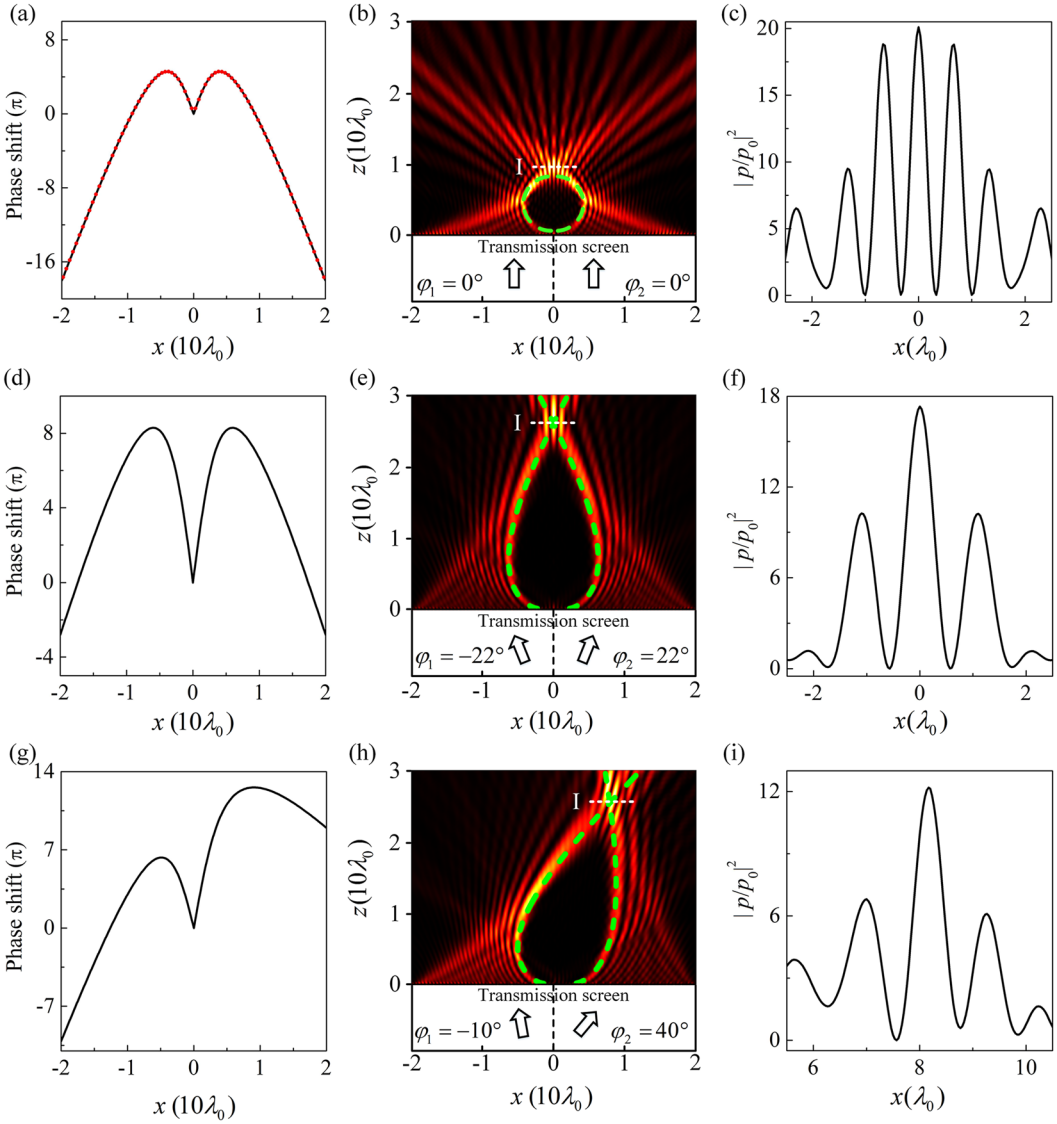
(**a**) Spatial phase profile in the *x*-axis for a circular acoustic bottle-beam and (**b**) the acoustic field distribution of the circular acoustic bottle-beam through the AM. Here, the incident angles *φ*_1_ = *φ*_2_ = 0° and the AM provides two mirror-imaged phase profiles in two sides of *x*-axis. (**c**) The intensity contrast ${|p/{p}_{0}|}^{2}$ profile on the line “I” in (**b**). The red dots in (**a**) represent the discrete phase profile of the subunits (one dot corresponds to one subunit). (**d**) Spatial phase profile in the *x*-axis for the case of (*φ*_1_ = −22°, *φ*_2_ = 22°) and (**e**) the corresponding acoustic field distribution of the acoustic bottle-beam. (**f**) The intensity contrast ${|p/{p}_{0}|}^{2}$ profile on the line “I” in (**e**). (**g**) Spatial phase profile in the *x*-axis for the case of (*φ*_1_ = −10°, *φ*_2_ = 40°) and (**h**) the corresponding acoustic field distribution of the acoustic bottle-beam. (**i**) The intensity contrast ${|p/{p}_{0}|}^{2}$ profile on the line “I” in (**h**). The green dashed lines indicate the theoretical propagation trajectories.

Figure 2(a) shows the spatial phase profile (solid line) in the *x*-axis according to Eq. (5). It is observed that two mirror-imaged phase profiles appear in two sides of *x*-axis. According this spatial phase profile, we design 80 TAS subunits with different *t*-values, and each subunit could provide the required transmitted phase [red dots in Fig. 2(a)] and ensure high transmission amplitude. The 40 subunits placed along negative *x*-axis provide mirror-imaged phases as those of 40 subunits in the positive *x*-axis. As an acoustic plane wave normally impinges on the AM from negative *z*-axis, a transmitted circular acoustic bottle-beam emerges from the upper surface of the AM, as shown in Fig. 2(b). The green dashed line represents theoretical circular trajectory, which matches well with the main lobe trajectory of simulation results. It is observed that most energy is focused into a focal region at the intersection of two accelerating beams. For a better view of the acoustic focusing, the intensity contrast ${|p/{p}_{0}|}^{2}$ near the focusing point [on the line “I” in Fig. 2(b)] is shown in Fig. 2(c). Here, *p* and *p*_0_ are the amplitudes of the transmitted and incident waves, respectively. The maximal intensity appears at the intersection of the two beams and the corresponding ${|p/{p}_{0}|}^{2}$-value could reach about 20, which is much better than those reported in some previous works^22,23^.

Then, we further give an additional gradient phase by changing the incident angle. The additional phase profiles are

6 $$\{\begin{array}{c}\varphi ^{\prime} (x,{\phi }_{1})=k\cdot \,\sin ({\phi }_{1})\cdot |x|,(x\le 0)\\ \varphi ^{\prime} (x,{\phi }_{2})=k\cdot \,\sin ({\phi }_{2})\cdot |x|,(x\ge 0)\end{array},$$

where *φ*_1_ and *φ*_2_ are the incident angles of the plan waves at negative and positive *x*-axis, respectively. || is the absolute value operator. In this case, Eq. (1) is revised as

7 $$\{\begin{array}{c}{{\rm{\Phi }}}^{-}(x)={\varphi }^{-}(x)+k\cdot \,\sin ({\phi }_{1})\cdot |x|,(x\le 0)\\ {{\rm{\Phi }}}^{+}(x)={\varphi }^{+}(x)+k\cdot \,\sin ({\phi }_{2})\cdot |x|,(x\ge 0)\end{array},$$

Eq. (3) should be revised as

8 $$\{\begin{array}{c}\frac{d{{\rm{\Phi }}}^{-}(x)}{dx}=-k\frac{{|x|}^{2}-{a}^{2}}{{|x|}^{2}+{a}^{2}}+k\cdot \,\sin ({\phi }_{1})=\frac{-k\,\tan (\theta )}{\sqrt{1+{\tan }^{2}(\theta )}},(x\le 0)\\ \frac{d{{\rm{\Phi }}}^{+}(x)}{dx}=-k\frac{{|x|}^{2}-{a}^{2}}{{|x|}^{2}+{a}^{2}}+k\cdot \,\sin ({\phi }_{2})=\frac{-k\,\tan (\theta )}{\sqrt{1+{\tan }^{2}(\theta )}},(x\ge 0)\end{array},$$

Figure 2(d) depicts the spatial phase profile in the *x*-axis for the case with (*φ*_1_ = −22° and *φ*_2_ = 22°). Due to two symmetric incident angles, the mirror-imaged phase profiles still appear in two sides of *x*-axis, but an outstretched acoustic bottle-beam is realized [green dashed line in Fig. 2(e)]. Figure 2(e) shows the transmitted acoustic fields through the AM based on FEM. Two incident acoustic plane waves propagate through the AM in negative *x*-axis with *φ*_1_ = −22° and in positive *x*-axis with *φ*_2_ = 22°, respectively, and an outstretched bottle-beam is observed. It is obvious that the main lobe trajectories of simulation results are in accord with the theoretical trajectories. Figure 2(f) shows the intensity contrast ${|p/{p}_{0}|}^{2}$ on the line “I” in Fig. 2(e). At the focusing point, the maximal ${|p/{p}_{0}|}^{2}$-value reaches ~17.3. Figure 2(g) shows the spatial phase profile of the AM for the case with *φ*_1_ = −10° and *φ*_2_ = 40°. Under this condition, an oblique bottle-beam can be achieved in Fig. 2(h). The maximal ${|p/{p}_{0}|}^{2}$-value at the focusing point reaches ~ 12.2, as show in Fig. 2(i).

Next, we generate the deflected acoustic bottle-beam based on the AM with asymmetric phase profiles in two sides of *x*-axis. For example, two parabolic trajectories are chosen: $x=-\,A{z}^{\frac{1}{2}}(x\le 0)$ and $x=-\,B{z}^{2}\,\,\,\,(x\ge 0)$. The corresponding phase profiles are

9 $$\{\begin{array}{c}{\varphi }^{-}(x)=k\frac{{A}^{2}}{4}[\mathrm{ln}(\sqrt{{x}^{2}+{(\frac{{A}^{2}}{4})}^{2}}-x)]\,,(x\le 0)\\ {\varphi }^{+}(x)=\frac{k}{2B}[\frac{1}{2}\,\mathrm{ln}(2\sqrt{Bx}+\sqrt{1+4Bx})-\sqrt{Bx(1+4Bx)}]\,,(x\ge 0)\end{array},$$

**Figure 3:**
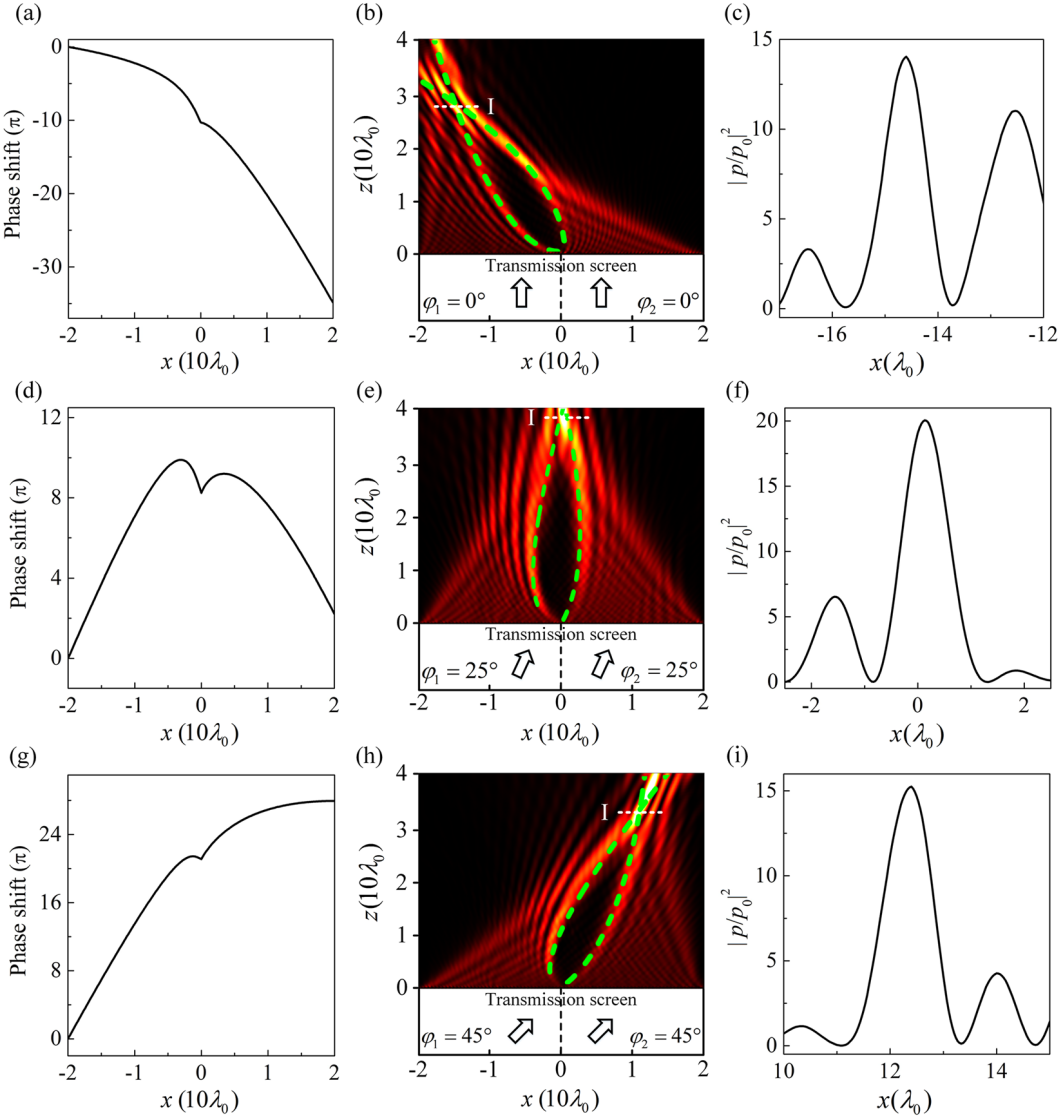
(**a**) Spatial phase profile in the *x*-axis for a deflected acoustic bottle-beam and (**b**) the acoustic field distribution of the deflected acoustic bottle-beam through the AM. Here, the incident angles *φ*_1_ = *φ*_2_ = 0°. (**c**) The intensity contrast ${|p/{p}_{0}|}^{2}$ profile on the line “I” in (**b**). (**d**) Spatial phase profile in the *x*-axis for the case of ${\phi }_{1}={\phi }_{2}={25}^{\circ }$ and (**e**) the corresponding acoustic field distribution of the acoustic bottle-beam. (**f**) The intensity contrast ${|p/{p}_{0}|}^{2}$ profile on the line “I” in (**e**). (**g**) Spatial phase profile in the *x*-axis for the case of (${\phi }_{1}={\phi }_{2}={45}^{\circ }$) and (**h**) the corresponding acoustic field distribution of the acoustic bottle-beam. (**i**) The intensity contrast ${|p/{p}_{0}|}^{2}$ profile on the line “I” in (**h**). The green dashed lines indicate the theoretical propagation trajectories.

Figure 3(a) shows the spatial phase profile of the AM based on Eq. (9). Here, *A* = 0.8 and *B* = 0.2. According to this phase profile, 80 TAS subunits have been well designed for the AM and are placed in two sides of *x*-axis. In Fig. 3(b), an acoustic plane wave propagates normally through the AM, whereupon a deflected acoustic bottle-beam emerges from the upper surface of the AM. The main-lobe trajectories of simulation results are in accord with the theoretical parabolic trajectories [green dashed line in Fig. 3(b)]. Figure 3(c) represents the intensity contrast ${|p/{p}_{0}|}^{2}$ on the line “I” in Fig. 3(b). The maximal ${|p/{p}_{0}|}^{2}$-value could reach about 14.

We further give an additional gradient phase by changing the incident angle. Substitute Eq. (9) into Eq. (3), we can get

10 $$\{\begin{array}{c}\frac{d{\Phi }^{-}(x)}{dx}=k(\frac{{A}^{2}}{4}\frac{1}{\sqrt{{x}^{2}+{(\frac{{A}^{2}}{4})}^{2}}})+k\cdot \,\sin ({\phi }_{1})\,=\frac{-k\,\tan (\theta )}{\sqrt{1+{\tan }^{2}(\theta )}},(x\le 0)\\ \frac{d{\Phi }^{+}(x)}{dx}=\frac{-2k\sqrt{Bx}}{\sqrt{1+4Bx}}+k\cdot \,\sin ({\phi }_{2})\,=\frac{-k\,\tan (\theta )}{\sqrt{1+{\tan }^{2}(\theta )}},(x\ge 0)\end{array},$$

Figure 3(d) depicts the spatial phase profile in the *x*-axis according to Eq. (10) for the incident angle *φ*_1_ = *φ*_2_ = 25°. By combining Eqs (4) and (10), we can obtain the numerical solution of the acoustic bottle-beam trajectory [green dashed line in Fig. 3(e)]. Figure 3(e) shows the transmitted acoustic fields through the AM with 80 TAS subunits for the incident angle *φ*_1_ = *φ*_2_ = 25° and the bottle-beam shows a clockwise rotation compared with the case of *φ*_1_ = *φ*_2_ = 0. The theoretical trajectories match well with the main lobe trajectories of simulation results. Figure 3(f) shows the intensity contrast ${|p/{p}_{0}|}^{2}$ on the line “I” in Fig. 3(e). The maximal ${|p/{p}_{0}|}^{2}$-value of ~ 20 appears at the focusing point. If the incident angles *φ*_1_ and *φ*_2_ both further increase to 45°, the bottle beam rotate clockwise a larger angle, as shown in Fig. 3(h). In Fig. 3(i), the maximal ${|p/{p}_{0}|}^{2}$-value at the focusing point is about 15.3.

**Figure 4:**
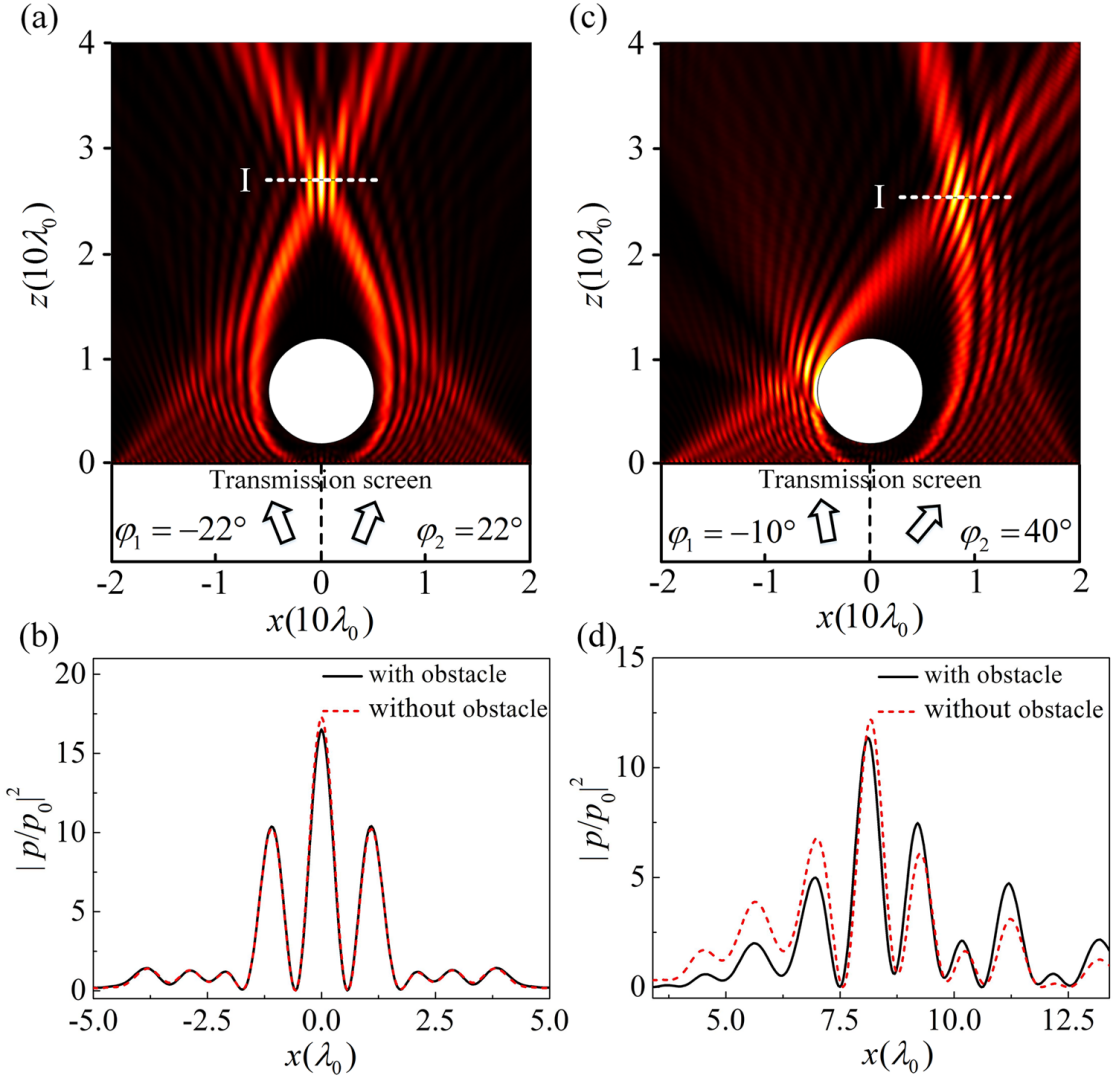
(**a**) Acoustic field distributions of the bottle-beams through the AM with an obstacle for incident angles (${\phi }_{1}=-\,{22}^{\circ }$,${\phi }_{2}={22}^{\circ }$) and (**b**) the intensity contrast ${|p/{p}_{0}|}^{2}$ profiles on the line “I”. (**c**) Acoustic field distributions of the bottle-beams through the AM with an obstacle for incident angles (${\phi }_{1}=-{10}^{\circ }$, ${\phi }_{2}={40}^{\circ }$) and (**d**) the intensity contrast ${|p/{p}_{0}|}^{2}$ profiles on the line “I”.

As shown in Figs 2 and 3, the AM generate two intersecting accelerating beams along the designed convex trajectories, which also can form a focus at the point of convergence. The position of the focus can be flexible controlled by changing the incident angles. Especially, such acoustic bottle-beam focusing is robust against the scattering from the possible obstacle inside the bottle. For example, we place a circular obstacle with radius 0.5*λ*_0_ in the acoustic bottle of Fig. 2(e). The center of the obstacle is at the point of (*x* = 0, *z* = 7*λ*_0_). As shown in Fig. 4(a), the acoustic field distribution with the obstacle is almost the same as that in Fig. 2(e), which means that the obstacle in the dark region of the acoustic bottle does not perturb the propagation of the beams. For a better view of the acoustic bottle-beam focusing, the focus intensity contrast ${|p/{p}_{0}|}^{2}$ near the focal point [on the dashed lines “I” in Fig. 4(a)] is shown in Fig. 4(b). The solid and dashed lines represent ${|p/{p}_{0}|}^{2}$-values for the case with and without an obstacle, respectively. It is found that the intensity peak nearly remain unchanged with an obstacle. Meanwhile, the intensity contrast reaches ~16. In Fig. 4(c), the acoustic bottle-beam focusing remains relatively stable even if we change the incident angles of *φ*_1_ = −10° and *φ*_2_ = 40°. In this case, the influence of the obstacle is still very weak and the ${|p/{p}_{0}|}^{2}$-value can reach ~12.5.

## Discussion

In summary, we designed an AM implemented by TAS subunits to generate acoustic bottle-beams. The acoustic bottle-beams are realized by two counter-accelerating beams with arbitrary convex trajectories. Caustic theory and geometrical properties are utilized to construct the relationship between an arbitrary bottle trajectory and the phase profile of the AM and FEM has been used to demonstrate the theoretical results. We find that the shape and area of the bottle-beams are statically controlled by designing the AM as well as dynamically modulated by the incident angles. In addition, the numerical simulation results show that the acoustic bottle-beam can generate high efficient acoustic focusing. Especially, this acoustic bottle-beam focusing could circumvent the obstacle and the focus can be controlled easily by changing the incident angle. We believe that the acoustic bottle-beams based on AM may be useful in biomedical imaging/therapy and non-destructive evaluation.

## Method

The numerical simulations are performed by using finite element method (FEM) based on COMSOL Multiphysics 5.2a software. The working frequency is fixed at 3.432 kHz and the background medium is air, the mass density and sound speed of it is *ρ*_*a*_ = 1.21 kg/m^3^ and *c*_*a*_ = 343.2 m/s. The material of the AM in simulation is chosen to be steel, whose mass density *ρ*_*s*_ = 7800 kg/m^3^ and acoustic speed *c*_*s*_ = 6100 m/s, respectively. Perfectly matched layers (PMLs) are utilized to eliminate the reflected waves by the outer boundaries. In all simulations, to ensure numerical accuracy, the largest mesh element size is lower than one tenth of the incident wavelength, and the further refined meshes are applied in the domain of the unit cells of the microstructure.
